# Cancer stem cells in brain tumors: From origin to clinical implications

**DOI:** 10.1002/mco2.341

**Published:** 2023-08-09

**Authors:** Shuyun Lin, Kaishu Li, Ling Qi

**Affiliations:** ^1^ Institute of Digestive Disease The Sixth Affiliated Hospital of Guangzhou Medical University Qingyuan People's Hospital Qingyuan Guangdong China

**Keywords:** brain tumors, cancer stem cells, cell of origin, clinical implications, immune system, stemness regulators, targeted therapies, tumor microenvironment

## Abstract

Malignant brain tumors are highly heterogeneous tumors with a poor prognosis and a high morbidity and mortality rate in both children and adults. The cancer stem cell (CSC, also named tumor‐initiating cell) model states that tumor growth is driven by a subset of CSCs. This model explains some of the clinical observations of brain tumors, including the almost unavoidable tumor recurrence after initial successful chemotherapy and/or radiotherapy and treatment resistance. Over the past two decades, strategies for the identification and characterization of brain CSCs have improved significantly, supporting the design of new diagnostic and therapeutic strategies for brain tumors. Relevant studies have unveiled novel characteristics of CSCs in the brain, including their heterogeneity and distinctive immunobiology, which have provided opportunities for new research directions and potential therapeutic approaches. In this review, we summarize the current knowledge of CSCs markers and stemness regulators in brain tumors. We also comprehensively describe the influence of the CSCs niche and tumor microenvironment on brain tumor stemness, including interactions between CSCs and the immune system, and discuss the potential application of CSCs in brain‐based therapies for the treatment of brain tumors.

## Graphical abstract

During tumorigenesis, the stemness of brain cancer stem cells is determined by diverse intrinsic and extrinsic forces that function simultaneously or independently over time. Intrinsic regulators include epigenetic regulators, transcription regulators, kinases, secretory molecules, metabolism regulators, other regulators and signaling pathways, while extrinsic regulators include interaction with the microenvironment, including niche factors and stromal cell

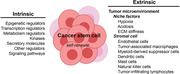

## 1 INTRODUCTION

Malignant brain tumors include primary tumors that originate from cells within the brain and metastatic tumors that spread to the brain from cancerous cells that originated in other parts of the body.^1^ Around half of malignant brain tumors are glioblastomas (GBMs), and 30% are diffusely infiltrating lower‐grade gliomas. Other malignant brain tumors include primary central nervous system lymphoma, malignant ependymomas, malignant meningiomas, and medulloblastoma (MB).^1^, ^2^, ^3^, ^4^ Although many cancer therapies have been developed over the past few decades, few drugs have been approved by the United States Food and Drug Administration (US FDA) for the treatment of brain tumors due to the difficulties in crossing the blood‒brain barrier (BBB). The prognosis for brain tumors has barely improved in recent decades. The current standard of medical care is maximal surgical resection with adjuvant chemotherapy and radiation therapy. One study reported that the median survival for the most aggressive GBM patients was only 14.6 months,^5^ and the 5‐year overall survival (OS) was <5%.^6^ Due to its high mortality rate and the limitations of existing treatment options, brain tumors is recognized as a public health problem.

Brain tumors is also a highly heterogeneous tumor. It is composed of a variety of cells with different molecular characteristics and different sensitivities to treatment. This heterogeneity not only underlies treatment resistance but also has broad implications for cancer therapy and biomarker discovery. There is now compelling evidence that intratumoral heterogeneity is driven by a subset of cells with stem or progenitor cell characteristics, termed cancer stem cells (CSCs). CSCs are a small population that is thought to be preserved by self‐renewal and has the ability to produce more differentiated progenies that make up the bulk of the tumor mass.^7^ However, the cell of origin for CSC generation has not been definitively identified, and almost certainly varies between different malignancies and possibly even between individual tumors of the same histology.^8^ In addition to providing a driving force for tumor growth, CSCs are considered to be the source of tumor recurrence and treatment resistance.^9^, ^10^, ^11^ Therefore, some commonalities of CSCs, such as self‐renewal, tumor initiation, tumor heterogeneity, and drug resistance, and some differences, such as origin, marker expression, differentiation potential, and tumor aggressiveness, can be observed in brain tumors.

This review summarizes the current knowledge of CSCs markers in the context of brain tumors and discusses the specific origin of brain CSCs, and, furthermore, focuses on intrinsic regulators of cancer stemness and effects of the tumor microenvironment in brain tumors. We also emphasis on the CSCs–immune system interactions, and the therapies targeting CSCs in brain that have potential value in the application development field.

## 2 THE ORIGIN OF CSCs

Carcinogenesis is a multistage process involving multiple mechanisms that mainly includes three stages.^12^ The first stage involves the irreversible conversion of a single normal cell into a premalignant or an initiated cell. The second step involves the amplification of a single initiated clone by mitogenesis, inhibition of apoptosis, or both. The third step involves a single initiated cell within the clone of promoted initiated cells accumulating other changes needed to confer the cell with all the hallmarks of cancer. To explain carcinogenesis, there are two opposing hypotheses for the origin of cancers. One hypothesis claims that an adult stem cell is target cell for initiating the carcinogenic process, whereas the other states that a somatic differentiated cell can dedifferentiate or be reprogrammed to regain cancer‐related properties. However, a major question remains: what causes normal cells to become cancerous? Regarding brain tumors, the cells of origin for CSCs include (1) adult neural stem cells (NSCs) acquire oncogenic mutations and (2) mature glial cells or restricted neural progenitor cells (usually terminally differentiated after successive divisions) that dedifferentiate to acquire unregulated stem cell‐like properties.^13^, ^14^, ^15^, ^16^

### 2.1 NSCs acquire oncogenic mutations to transform into CSCs

Many studies have reported that the introduction of oncogenic mutations in NSCs can induce cancer formation. However, the mechanism by which NSCs transform into CSCs and lead to tumor formation is still unclear. Studies have shown that various conditions, including tissue damage, radiation therapy, and exposure to toxins (such as smoking), can cause mutations in certain genes. Prolonged continuous division of stem cells also increases the chance of accumulating mutations. Mutations in oncogenes may cause NSCs to transform into CSCs. Wang et al.^17^ showed that introduction of GBM‐associated mutations in tumor suppressors (*tumor protein p53* [*TP53]*/*neurofibromatosis type 1* [*NF1*]/phosphatase and tensin homolog [*PTEN*] or *TP53*/*NF1*) into human NSCs generates CSC populations and induces brain tumors. In addition, harmful external signals can deactivate or enhance certain signaling pathways in NSCs, thereby leading to the transformation of NSCs into CSCs. For example, GBM extracellular vesicles, which play an important role in the tumor microenvironment, can promote the transformation of NSCs in cancerous cells. The mechanism may be related to several key genes, such as S100 calcium binding protein B, C‐X‐C motif chemokine ligand 14 (CXCL14), EGF‐containing fibulin extracellular matrix (ECM) protein 1, stimulator of chondrogenesis 1, GLI pathogenesis‐related 1, high‐mobility group AT‐hook 1, and CD44, and dysregulated signaling.^18^ Abnormal expression of epigenetic molecules in NSCs can also lead to the formation of CSCs. Landskron et al.^19^ demonstrated that high expression of the lncRNA cherub in NSCs leads to the formation of CSCs.

### 2.2 Dedifferentiation of glial cells or neural progenitor cells from CSCs

Studies have shown that chromatin‐related regulators can induce the formation of CSCs. Sequencing studies have identified a high frequency of histone H3 mutations in childhood glioma.^20^, ^21^ The gene mainly affected is histone 3 variant H3.3, and the K27M substitution is the most common alteration. In neural progenitor cells derived from human embryonic stem cells, H3.3K27M expression synergizes with p53 loss and platelet‐derived growth factor receptor alpha activation to lead to neoplastic transformation. Further investigation revealed that the mechanism may be related to the expression of the H3.3K27M mutant leading to a developmental resetting of neural precursor to a more primitive stem cell state.^22^ However, the role of H3.3 in adult GBM appears to be different. In adult GBM, H3.3 is suppressed in self‐renewing GBM cells, and its overexpression antagonizes self‐renewal and promotes differentiation,^23^ suggesting that the mechanism by which glioma stem cells (GSCs) are formed in adult GBM is different from that in childhood glioma. Furthermore, lacking PTEN and TP53 in Olig1/2‐expressing intermediate lineage progenitors induces activation of oncogenic pathways, including PI3K and HIPPO–YAP signaling, thereby promoting malignant transformation and gliomagenesis.^24^

In addition, members of the inhibitor of differentiation (ID) family (ID1–ID4), which are cell fate determinants, can also induce the formation of CSCs. For example, ID3 overexpression imparts GSC features to primary astrocytes derived from Ink4a/Arf‐deficient mice.^25^ Overexpression of ID4 drives astrocytes into a neural stem‐like cell state by activating Notch signaling.^26^ Although many studies have reported that the dedifferentiation of glial cells or neural progenitor cells can induce the formation of CSCs, the molecular mechanism of the dedifferentiation of glial cells or neural progenitor cells into CSCs is still unclear.

## 3 MARKERS OF CSCs IN BRAIN TUMOR

**FIGURE 1:**
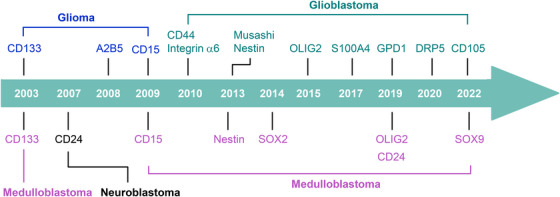
Cancer stemness‐related discoveries in brain tumor. Early studies identified various brain CSC markers.

Much of the early research on brain CSCs focused on identifying brain CSC markers derived from cell‐sorting assays and xenotransplantation experiments in immunodeficient mice (Figure 1 and Table 1). Although one should not overly rely on markers to identify CSCs, this early study undoubtedly laid the groundwork for current research on brain cancer stemness. Some candidate markers for identifying brain CSCs include oligodendrocyte transcription factor 2 (Olig2), SRY‐box transcription factor 2 (SOX2), nestin, S100 calcium‐binding protein A4 (S100A4), CD133, fucosyltransferase 4 (CD15), integrin α6, CD44, A2B5, CD24, Musashi, and CD105.^27^, ^28^, ^29^, ^30^, ^31^, ^32^, ^33^, ^34^, ^35^, ^36^, ^37^, ^38^, ^39^, ^40^, ^41^, ^42^, ^43^, ^44^, ^45^, ^46^, ^47^, ^48^, ^49^, ^50^, ^51^ However, most of these markers are also expressed in NSCs. Therefore, several studies have specifically analyzed brain CSCs‐specific markers. For example, glycerol‐3‐phosphate dehydrogenase 1 (GPD1) specifically marks dormant brain CSCs. GPD1 is specifically expressed in brain CSCs and not in NSCs.^52^ Dihydropyrimidinase‐related protein 5 (DRP5) was specifically upregulated in the proneural‐subtype GSCs and plays a key role in maintaining GSC properties.^53^ SOX9 is limited to rare and quiescent cells in high‐risk MB with MYC amplification. Dormant SOX9‐positive cells promote MYC‐driven recurrence of MB.^54^

**TABLE 1:** Markers of cancer stem cells in brain tumor.

Name	Non‐brain tumor cell types often associated with this marker	Function	Experimental evidence	References
Intracellular markers				
Olig2	Oligodendrocyte progenitor cells and motor neurons	A essential regulator of ventral neuroectodermal progenitor cell fate	Lineage‐tracing, lineage‐ablation studies, single‐cell sequencing	27, 28, 29, 30
Musashi	Glial and neuronal progenitor cells	mRNA binding protein that promotes down regulation of 26S proteasome. Inhibition of mRNA translation	NA	50
SOX2	Embryonic stem cells and neural tubes	A member of the family of transcriptional cofactors that plays a role in maintaining pluripotency	Lineage‐tracing	31, 32, 33, 34
Nestin	Neuronal stem cells	Proliferation/migration	FACS	35, 36
S100A4	Glial cells, satellite cells and neurons	An intracellular calcium‐binding protein that interacts to other proteins to enhance apoptosis, cell motility and angiogenesis	FACS, lineage‐ablation studies	37
Cell surface markers				
CD133	Hematopoietic, neural stem cells, adult ependymal cells, and endothelial precursor cells	A transmembrane glycoprotein that maintains lipid composition in cell membranes	FACS, xenotransplantation	38, 39
CD15	Embryonic and adult neural stem/progenitor cells	A Cluster of differentiation antigen and carbohydrate adhesion molecule regulate cell proliferation, self‐renewal, and multilineage differentiation	FACS, magnetic bead sorting, xenotransplantation	40, 41, 42
Integrin α6	Neural stem cell	A member of the integrin family of extracellular matrix receptors for laminin and platelets	FACS, xenotransplantation	43, 44
CD44	Mesenchymal cells	A glycoprotein involves in cell migration and self‐renewal	FACS, xenotransplantation	45
A2B5	Oligodendrocyte progenitor cells	A ganglioside marker that identifies subpopulations of nerve cells in the central nervous system	FACS, xenotransplantation	46, 47
CD24	Differentiating neuroblasts	A cell adhesion glycosylphosphatidylinositol anchor protein that plays an important role in neural migration, neurite outgrowth and neurogenesis	FACS	48, 49
CD105	Mesenchymal stem cells	A type I transmembrane protein belonging to the transforming growth factor beta receptor family that involves angiogenesis	FACS, xenotransplantation	51

NA, not available; FACS, fluorescence‐activated cell sorting.

It is noteworthy, some brain CSC markers are known to be associated with more aggressive brain tumor and with worse patient survival. For example, the prognostic significance was identified for CD133 and nestin in the majority of studies, and the coexpression of CD133 and nestin was shown to have better prognostic value than the single markers alone.^55^ High levels of Musashi‐1 were found to be associated with poor survival in WHO grade III disease, although the conclusion was made based on very few patients.^56^ However, the diversity and application value of CSC markers in the complex heterogeneous tumor microenvironment requires more systematic and in‐depth research.

## 4 INTRINSIC REGULATORS OF CANCER STEMNESS IN BRAIN TUMOR

**FIGURE 2:**
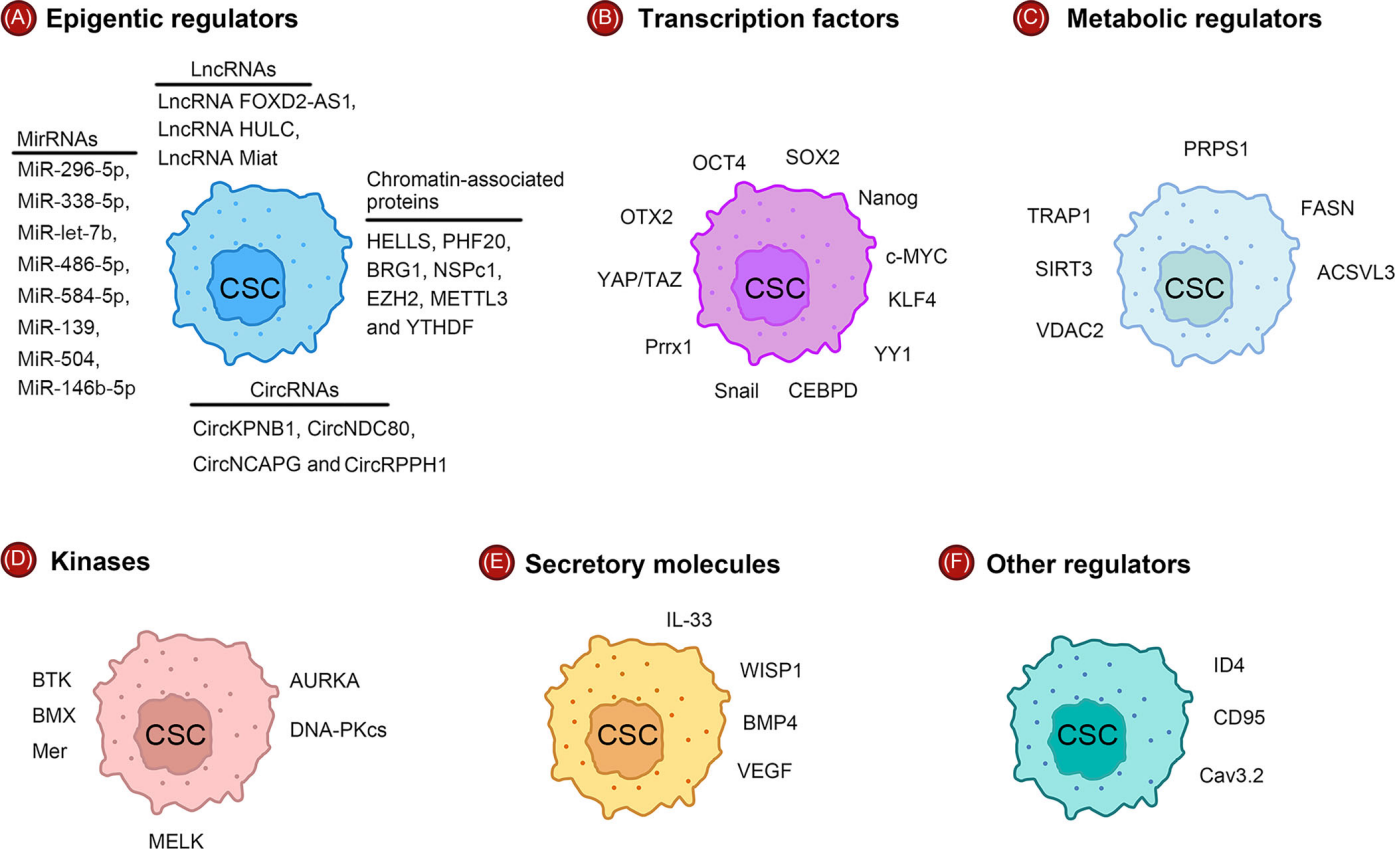
Intracellular forces regulate the stemness of CSC. Key intrinsic regulators include (A) epigenetic regulators, (B) transcription regulators (C) metabolism regulators, (D) kinases, (E) secretory molecules, and (F) other regulators.

The acquisition of uncontrolled self‐renewal is only the first step in the development of cancer. Malignantly transformed cells require the ability to self‐renew as the cancer grows. Many intrinsic and extrinsic regulators are known to be important for maintaining the uncontrolled self‐renewal and tumor‐initiating potential of CSCs (Figure 2).

### 4.1 Epigenetic regulators

As a well‐recognized cancer hallmark, epigenetic alterations are driven by epigenetic regulators. That a wide range of epigenetic regulators can be influenced by driver mutations in hierarchically organized cancers provides important direct evidence for a role of epigenetic dysregulation in the formation of CSCs.^57^ Given that epigenetic regulators is important for the maintenance of NSCs, it is not surprising that many chromatin‐associated proteins are essential for maintenance of the CSCs state as well as tumorigenesis. Helicase, lymphoid specific (HELLS), a chromatin‐remodeling factor, is preferentially expressed in GSCs. Targeting HELLS disrupts GSCs proliferation, survival, and self‐renewal by inducing replication stress and DNA damage, thereby inhibiting GBM growth.^58^ Plant homeodomain finger‐containing protein 20 (PHF20) can interact with poly(ADP‐ribose) polymerase 1 (PARP1) and directly binds to promoter regions of stemness genes to regulate the levels of OCT4 and SOX2, thereby sustaining stem cell‐like phenotype of neuroblastoma.^59^ In addition, the catalytic subunit brahma related gene 1 (BRG1) of the SWI/SNF complex can maintain the stemness of glioma‐initiating cells by inhibiting the STAT3/TXNIP pathway, resulting in the resistance of glioma‐initiating cells to the chemotherapeutic drugs temozolomide (TMZ) and carmustine.^60^ The polycomb repressive complex 1 (PRC1) subunit nervous system polycomb 1 (NSPc1) and the PRC2 subunit enhancer of zeste 2 (EZH2) have been found to inhibit GSCs self‐renewal and proliferation by reducing the expression of RDH16 and CD133, respectively.^61^, ^62^ In addition, it has been reported that some histone deacetylase (HDAC) inhibitors, such as the HDAC 3 inhibitor RGFP966, suberoylanilide hydroxamic acid, Domatinostat, and HDAC 6 inhibitors, can inhibit the proliferation and self‐renewal of GSCs and promote their dedifferentiation, suggesting that HDACs could be targets for drug therapy against glioma.^63^, ^64^, ^65^, ^66^

Noncoding RNAs (ncRNAs) are a group of RNAs that do not have coding functions. A large number of studies have proven that the expression of ncRNAs is heterogeneous in tumors. Some ncRNAs are specifically expressed in CSCs and participate in their functional regulation. There are many studies of the maintenance of brain stemness by miRNAs. MiRNAs can regulate brain CSCs stemness by regulating stemness‐related genes or transcription factors. For example, miR‐296‐5p modulates SOX2 expression,^67^ miR‐338‐5p binds to the FOXD1 3′‐UTR,^68^ miR‐let‐7b downregulates E2F2 expression,^69^ miR‐486‐5p targets PTEN and FoxO1,^70^ and miR‐584‐5p targets eIF4E3.^71^ MiRNA can also directly or indirectly regulate the signaling pathways associated with brain CSCs self‐renewal; for example, WNT‐β‐catenin signaling is regulated by the effects of miR‐139 on FZD3 and β‐catenin as well as by the effects of miR‐504 on FZD7.^72^, ^73^ Several lncRNAs have been found to be critical for stemness regulation in the brain. The lncRNA FOXD2 adjacent opposite strand RNA 1 (FOXD2‐AS1) is significantly upregulated in GSCs. Silencing LncRNA FOXD2‐AS1 impairs stemness and proliferation of GSCs, while promotes GSCs apoptosis and differentiation by inhibiting NOTCH signaling.^74^ Similarly, silencing of the lncRNA highly upregulated in liver cancer (lncRNA HULC) inhibited the stemness and proliferation of GSCs and promoted the apoptosis and differentiation of GSCs, thereby inhibiting the occurrence and development of glioma.^75^ In addition, the lncRNA Miat is required for maintenance of a treatment‐resistant stem‐like phenotype in MB. Absence of Miat leads to differentiation of tumorigenic stem‐cell like MB cells and differentiation of tumorigenic stem‐cell like MB cells into a non‐tumorigenic state.^76^ Most recently, circular RNAs has also been demonstrated to regulate pluripotency and tumor cell growth in brain tumor, such as circNDC80, circNCAPG, circRPPH1, and circKPNB1.^77^, ^78^, ^79^, ^80^ Taken together, these findings suggest that ncRNAs not only affect the function of non‐CSCs but also regulate the function of CSCs. Studying the effects of these ncRNAs on CSCs can provide a better understanding of brain tumor initiation and progression.

In addition to the abovementioned epigenetic regulators, m^6^A‐regulated proteins have also recently been linked to the maintenance of stemness in CSCs. For example, methyltransferase 3, N6‐adenosine‐methyltransferase complex catalytic subunit (METTL3) increases GSCs stemness by mediating m6A modification of SOX2 mRNA, thus maintaining SOX2 stability.^81^ YTH N6‐methyladenosine RNA binding protein 2 (YTHDF2) is essential for GSCs maintenance because it stabilizes MYC mRNA.^82^ Notably, most proteins involved in CSCs maintenance are usually not mutated so that cancer cells can avoid differentiation and maintain their malignant properties.

### 4.2 Transcription regulators

In addition to octamer‐binding transcription factor 4 (OCT4), SOX2, Nanog homeobox (Nanog), proto‐oncogene, bHLH transcription factor (c‐MYC), and KLF transcription factor 4 (KLF4), which are well‐known regulators of CSCs, some of the less‐studied transcription factors have also been reported to have a role in the maintenance of stem cell‐like features in brain tumor. Transcription factor Yin Yang 1 (YY1) can increase the m6A modification level of MYC mRNA by enhancing the methylase activity of METTL3, thereby promoting the self‐renewal of GSCs.^83^ CCAAT/enhancer‐binding protein delta (CEBPD) is proposed to be oncogenic given its relationship with drug resistance. CEBPD can increase the protein levels of SOX2, OCT4, and Nanog, thereby promoting GSCs formation and further contributing to TMZ resistance.^84^

In addition, snail family transcriptional repressor 1 (snail) promotes bone morphogenetic protein (BMP) signaling and subsequent cell differentiation and inhibits the survival and self‐renewal of GSCs by decreasing TGFβ1 expression and its downstream signaling.^85^ TGF‐β1 signaling is also controlled by the transcription factor paired‐related homeobox 1 (Prrx1), which directly binds to the promoter regions of the TGF‐β1 gene, upregulates the expression of TGF‐β1, and ultimately activates the TGF‐β/smad pathway, thereby increasing stemness acquisition in non‐stem tumor cells and maintaining stemness in GSCs.^86^ A recent single‐cell sequencing study revealed that the transcriptional coactivators YAP/TAZ act as the main roadblock of GSCs differentiation and that their inhibition irreversibly locks differentiated GBM cells into a nontumorigenic state, preventing GSCs plasticity and regeneration.^87^ The transcription factor orthodenticle homeobox 2 (OTX2) inhibits differentiation and promotes self‐renewal and tumor initiation of MB stem/progenitor cells through semaphorin signaling.^88^, ^89^

### 4.3 Metabolism regulators

Research has shown that brain CSCs undergo a unique pattern of metabolic reprogramming that is critical for maintaining their stemness.^90^ Indeed, cancer cells undergo changes in metabolism, such as enhanced anaerobic glycolysis, because of upregulation of the transcription of genes related to glycolysis.^91^, ^92^ Metabolic reprogramming targeting CSCs may be a promising treatment for brain tumor.^93^, ^94^ The cooperative interplay between the major mitochondrial deacetylase sirtuin‐3 (SIRT3) and the mitochondrial chaperone TNF receptor‐associated protein 1 (TRAP1) in GSCs has been found to increase the respiratory capacity mitochondrial and reduce the generation of reactive oxygen species. This metabolic regulation endows GSCs with metabolic plasticity and maintains stemness. Inactivation of SIRT3 or TRAP1 disrupts their interdependent regulatory mechanisms, resulting in metabolic alterations, loss of stemness, and inhibition of tumor formation by GSCs.^95^ Another protein located on mitochondria, voltage‐dependent anion channel 2 (VDAC2), was reported to be critical for the metabolic switch between GSCs and non‐stem tumor cells because it affects phenotype. Disrupting VDAC2 expression in non‐stem tumor cells promoted the activation of glycolytic metabolism, thus potentiating the acquisition of GSC properties. Forced expression of VDAC2 in GSCs suppressed glycolytic activity and disrupted GSC features.^96^ Combined metabolomic and genomic analyses revealed that phosphoribosyl pyrophosphate synthetase 1, which is a purine synthase and catalyzes the first step of de novo purine synthesis, promotes GSCs self‐renewal, growth and tumor formation in vivo.^97^ Another independent study has also shown that purine synthesis inhibition reduces GBM stemness through attenuated mitochondrial respiration and mitochondrial spare respiratory capacity.^98^

The brain CSC phenotype is also associated with the modulation of lipid synthesis by fatty acid synthase (FASN) and acyl‐CoA synthetase VL3 (ACSVL3). FASN, a key lipogenic enzyme, controls de novo synthesis of lipids. Inhibition of FASN reduces the expression of the GSC markers SOX2, nestin and fatty acid binding protein and increases the expression of glial fibrillary acidic protein, suggesting that FASN has a key role in the maintenance of GSCs stemness.^99^ ACSVL3 has been reported to be up‐regulated in malignant brain tumor tissues and is involved in tumorigenesis. Downregulating ACSVL3 decreased the expression of markers and regulators associated with stem cell self‐renewal, including CD133, Musashi‐1, SOX2, and aldehyde dehydrogenase, suggesting that ACSVL3 participates in the maintenance of GSCs and affects the tumor‐initiating capacity of GSCs.^100^

In addition, recent research has shown that isocitrate dehydrogenase (IDH) mutations have a significant impact on brain CSCs.^101^ IDH, an important metabolic enzyme in the tricarboxylic acid cycle, has been found to mutate frequently in brain tumors. Mutant IDH enzyme activity results in the conversion of α‐KG into 2‐hydroxyglutarate, which can promote hypermethylation of CpG islands and altered histone methylation.^102^, ^103^, ^104^ The global epigenomic reprogramming induced by IDH mutation cancer leads to differentiation arrest, upregulation of stem cell‐associated pathways and the development of a CD24‐positive cell population in gliomas.^101^, ^103^, ^105^, ^106^, ^107^, ^108^ Although recent studies have shed light on the research about IDH mutation and CSCs, many questions remain unanswered. For example, it is not clear how IDH mutations affect the differentiation and self‐renewal capacities of brain CSCs, or how these mutations alter the metabolic and epigenetic profiles of these cells. Overall, the interaction between IDH mutations and brain CSCs is complex and multifaceted, and further research is needed to fully understand the mechanisms involved.

### 4.4 Kinases

Kinases have attracted growing interest from researchers for their therapeutic potential. The cell cycle‐regulated kinase aurora kinase A (AURKA) has been reported to interact directly with AXIN, disrupt the AXIN/GSK3β/β‐catenin complex, and stabilize β‐catenin, thus activating Wnt signaling to promote the self‐renewal of glioma‐initiating cells.^109^ Blockade of AURKA signaling with alisertib induces significant antitumor activity in bevacizumab‐resistant patient‐derived orthotopic models of GBM.^110^ DNA‐dependent protein kinase catalytic subunit (DNA‐PKcs) was found to be preferentially expressed in GSCs. DNA‐PKcs can phosphorylate SOX2 at S251, which stabilizes SOX2, thereby promoting GSCs maintenance. Pharmacological inhibition of DNA‐PKcs attenuates GBM growth and sensitizes GBM to radiotherapy.^111^

In addition to protein kinases that regulate the cell cycle, other kinases have also been reported to affect the stemness of GSCs. Bruton's tyrosine kinase (BTK), a nonreceptor tyrosine kinase, plays a key role in GBM tumorigenesis and GSCs maintenance/generation by regulating CD133 and Akt/mTOR signaling. Inhibition of BTK by ibrutinib reduces GBM formation and affects the stem cell phenotype.^112^ Nonreceptor tyrosine kinase bone marrow X‐linked maintains the self‐renewal and tumorigenic potential of GSCs through activating STAT3 signaling.^113^ STAT3 signaling is also regulated by the receptor tyrosine kinase Mer to maintain GSCs self‐renewal.^114^ In addition, the serine/threonine kinase maternal embryonic leucine‐zipper kinase can bind to and phosphorylated EZH2, and the interaction of the two proteins can regulate the self‐renewal of sonic hedgehog subtype MB.^115^ Taken together, these findings demonstrate the important role of kinases in maintaining the uncontrolled self‐renewal of brain CSCs.

### 4.5 Secretory molecules

Some secreted molecules have also been reported to regulate CSC stemness. For instance, interleukin‐33 (IL‐33), activate the JNK signaling pathway through ST2 and increase the expression of key transcription factors that control stemness processes.^116^ The secreted protein Wnt‐induced signaling protein 1 (WISP1) maintains GSCs through integrin α6β1‐Akt signaling in an autocrine manner.^117^ BMP4 suppresses GSCs proliferation by downregulating cyclin D1 levels and promotes GSCs apoptosis by inducing Bax and inhibiting Bcl‐2.^118^ Another research team found that BMP4 can affect the lineage selection and phenotypic plasticity of GSCs.^119^ In addition, a series of studies have reported that vascular endothelial growth factor (VEGF) promotes the self‐renewal and growth of GSCs by mediating STAT3 activation^120^ and augmenting VEGF receptor 2 (VEGFR2) signaling cascades.^121^, ^122^ VEGFR2 inhibition reduces the self‐renewal capacity of GSCs, forming tubules and promoting vascularization and the establishment of vasculogenic mimicry.^123^

### 4.6 Other regulators

In addition to the abovementioned epigenetic regulators, transcriptional regulators, metabolic regulators, kinases, and secretory factors, many regulators that cannot be broadly classified into the above groups play an important role in the maintenance of brain CSCs stemness. For example, ID4 enhances SOX2 protein expression by inhibiting miR‐9*, thereby conferring stemness and chemotherapy resistance to glioma cells and GSCs.^124^ The expression of fas cell surface death receptor (CD95) is correlated with stemness in GBM tumors and cells and serves as a prognostic biomarker. The combination of TMZ with a CD95 inhibitor significantly abrogates tumor sphere formation.^125^ Inhibition of the T‐type calcium channel calcium voltage‐gated channel subunit alpha1 H inhibits GSCs growth by inhibiting the pro‐survival AKT/mTOR pathway and stimulating the pro‐apoptotic survivin and BAX pathways.^126^

### 4.7 Signaling pathways

Despite the fact that we have described a range of regulators that regulate brain tumor stemness, it is clear that many of them converge into commonly altered signaling pathways. These signaling pathways include the Wnt‐β‐catenin and Notch pathways. Wnt‐β‐catenin signaling is promoted by overexpression of solute carrier family 34 member 2, overexpression of human tripartite motif 59, and inhibition of period circadian clock 2.^127^, ^128^, ^129^ Notch signaling is promoted by overexpression of transient receptor potential melastatin‐related 7, ligand of numb protein 1, and lysine demethylase 5B.^130^, ^131^, ^132^ In addition, cyclophilin A and arsenite‐resistance protein 2 have also been demonstrated to increase the stemness properties of GSCs by activating WNT‐β‐catenin signaling.^133^, ^134^ STAT3 signaling is also frequently altered in brain CSCs and is required for brain CSCs to proliferate and maintain pluripotency.^135^ CD109 physically interacts with glycoprotein 130 to promote IL‐6/STAT3 pathway activation, which drives GSCs plasticity and chemoresistance.^136^ Similar findings were observed in GBM; RBPJ can increase GBM cell proliferation, invasion, stemness, and tumor initiating ability by enhancing the activation of the IL‐6/STAT3 pathway.^137^ In addition, insulin‐like growth factor 1 receptor regulates CSC‐like properties in NB cells via the activation of the STAT3/AKT pathway.^138^

## 5 EFFECTS OF THE TUMOR MICROENVIRONMENT IN BRAIN TUMOR

**FIGURE 3:**
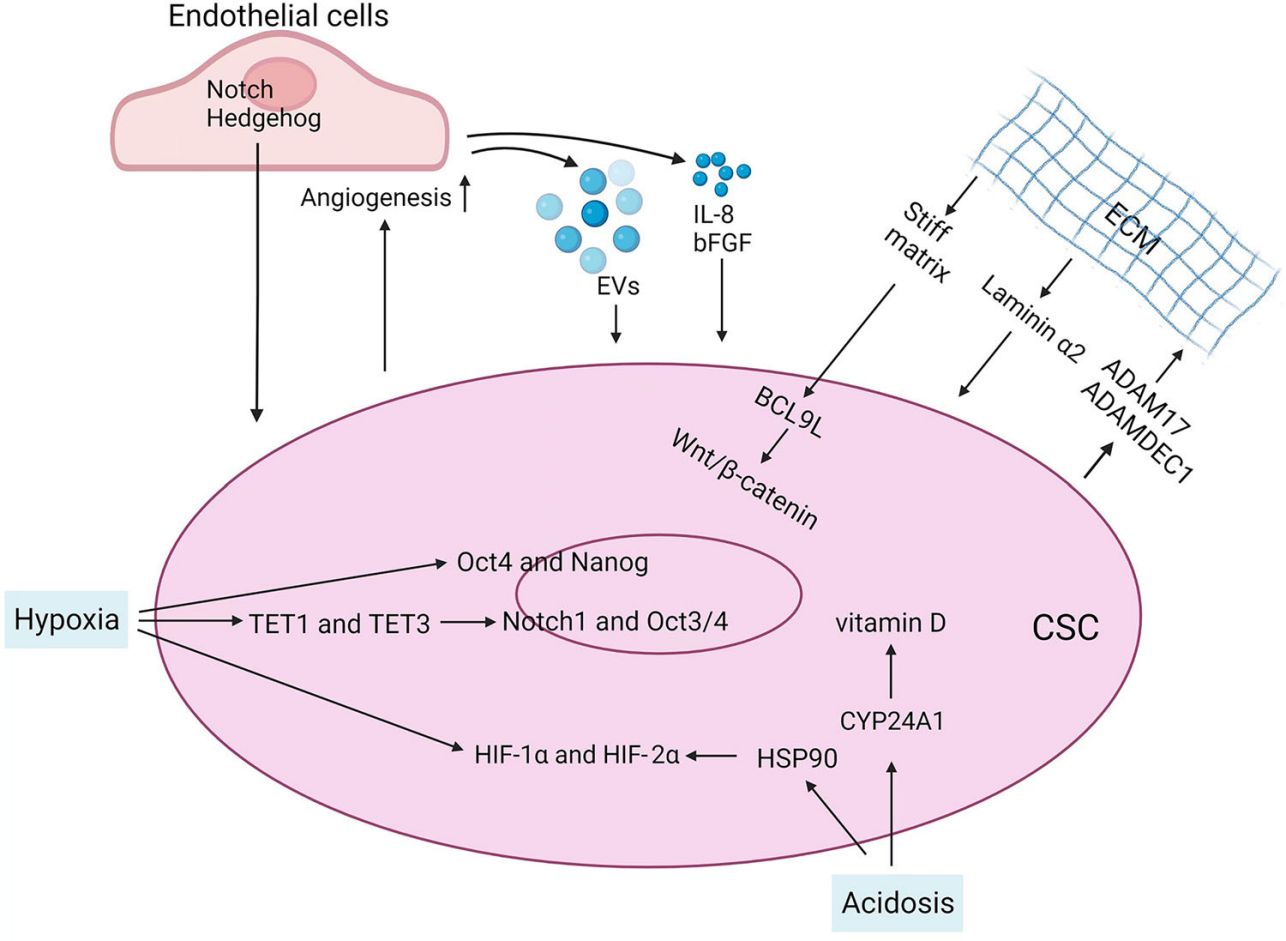
Tumor microenvironmental influences on CSCs. The tumor‐associated stroma has an important role in the regulation of cancer stemness in glioma. Regulation of CSCs by their niche occurs through cell–matrix interactions and cell–cell interactions. The most prominent players in glioma CSC–stroma interactions include hypoxia, acidosis and extracellular matrix (ECM) remodeling; cellular components include endothelial cells.

Stem cells of normal tissues exist in a specific microenvironment called the stem cell niche, which consists of a variety of stromal cells (such as immune cells and mesenchymal), vascular networks, components of the ECM, and soluble factors. Similar to normal stem cells, CSCs also appear to depend on a similar environment, termed the CSC niche, which maintains the unique ability of CSCs to self‐renew and produce more differentiated progenitors while remaining undifferentiated themselves.^139^, ^140^, ^141^ In this section, we focus on the most important factors involved in CSCs–stroma interactions (Figure 3). These factors include hypoxia, acidosis, ECM stiffness, and endothelial cells (ECs).

### 5.1 CSCs and hypoxia

Hypoxia is a common characteristic of the tumor microenvironment that promotes the proliferation of cancer cells by overcoming the limitations of blood supply. Hypoxia is known to promote CSC stemness.^142^, ^143^ Earlier studies have found that hypoxia increases the expression of CD133, SOX2, OCT4, and nestin in CD133‐positive GBM cells and increases the self‐renewal capacity of CD133‐positive GBM cells.^144^ Another study showed that hypoxia increases the expression of OCT4 by inducing the expression of the demethylases TET1 and TET3, thereby promoting the stemness of glioma cells.^145^ Hypoxia can also promote GSCs proliferation and maintain stem cells characteristics via activating Notch1 and OCT3/4, thus affecting the biological characteristics of glioma cells.^146^ In addition, hypoxia‐inducible factor 1 alpha (HIF‐1α) and HIF2α were found to be critical for the function of CSCs.^146^, ^147^, ^148^, ^149^ Stabilization of HIF1α leads to expansion of the GSCs population in tumors. Conversely, silencing HIF1α abolishes the self‐renewal capacity of GSCs and results in reduced tumorigenic potential in vivo.^150^ HIF1α/HIF2α induce the dedifferentiation of glioma cells into CSCs through SOX2 under hypoxic conditions, thereby promoting chemoresistance of glioma cells.^151^ Other evidence also supports that differentiated GBM cells undergo dedifferentiation under hypoxic conditions to generate GSCs.^152^ Simultaneous knockdown of HIF1α and HIF2α inhibits cell cycle arrest but promotes the proliferation and chemosensitization of GBM cells while reducing their stemness.^152^ Taken together, these findings demonstrate that hypoxia and HIFs exert an important influence in maintaining the GSCs phenotype through various mechanisms.

### 5.2 CSCs and acidosis

One of the hallmarks of the brain tumor microenvironment is low pH. Normal brain tissue has been reported to have a pH of approximately 7.1, and solid tumors, including GBM, have an average extracellular pH of 6.8.^142^ Acidosis can act synergistically with hypoxia to upregulate HIFs and GSCs maintenance in glioma through HSP90.^153^ Genetic or pharmacological inactivation of HSP90 suppresses the increase in HIF levels and abrogates the self‐renewal and tumorigenic properties of acidosis‐induced CSCs.^153^ Acidic stress can also promote the GSCs phenotype independently of hypoxia, but the mechanism may still involve HIF2α expression. For example, low pH leads to the expression of HIF2α and HIF target genes, promoting the CSC phenotype.^154^ Importantly, acid stress can also affect cellular metabolism. It has been shown that GSCs cultured under low pH conditions exhibit increased de novo purine nucleotide biosynthesis.^155^ In addition, in an acidic microenvironment, 25‐hydroxy vitamin D3‐24‐hydroxylase (CYP24A1) is elevated to catalyze the rapid degradation of vitamin D, which in turn inhibits the expression of stem cell markers and attenuates the acidosis‐induced increase in self‐renewal ability and mitochondrial respiration of GSCs.^156^

### 5.3 CSCs and ECM stiffness

As an important component of the tumor microenvironment, the ECM is mainly composed of glycoproteins, glycosaminoglycans, and proteoglycans. It is both an extracellular scaffold and a dynamic compartment where components are constantly deposited, degraded, or remodeled. Changes in the ECM are not only critical for tissue structure but also affect several biological mechanisms. Indeed, an increasing number of studies have reported that the ECM stiffness of the tumor microenvironment plays an important role in regulating CSCs during tumorigenesis.^157^ CD133^+^ glioma cells express increased levels of A disintegrin and metalloproteinase 17 (ADAM17) and a disintegrin and metalloproteinase domain‐like protein decysin 1 (ADAMDEC1), two metalloproteinases that degrade ECM proteins and promote stemness in CSCs.^158^, ^159^ The ECM marker laminin α2 is expressed in the perivascular GSC niche, where it contributes to the maintenance of GSCs and protects GSCs from radiation‐induced damage.^160^ 3D porous scaffolds composed of different ECMs have been found to enrich GSCs and increase the expression of GSC biomarkers.^161^ Culturing glioma cells on stiff polyacrylamide hydrogels increased their proliferation and CD133 expression.^162^ Greater matrix stiffness significantly upregulated BCL9L expression, thereby activating Wnt/β‐catenin signaling and ultimately enhancing the stemness of glioma cells.^162^

### 5.4 CSCs and ECs

Brain CSCs are enriched in the perivascular niche where ECs are crucial to control CSC stemness.^163^, ^164^ It has been reported that ECs are responsible for regulating the growth and self‐renewal and maintaining the stemness of brain tumor cells through intracellular signaling pathways such as the Hedgehog and Notch pathways.^165^, ^166^, ^167^, ^168^, ^169^ ECs can also regulate the survival, growth, and self‐renewal of brain CSCs by secreting factors such as IL‐8 and bFGF, thereby promoting tumor growth.^163^, ^170^, ^171^, ^172^, ^173^, ^174^ ECs have also been found to promote GSCs growth and self‐renewal to enhance GBM invasiveness by releasing extracellular vesicles.^175^ In turn, brain CSCs can encourage ECs to form blood vessels through multiple mechanisms, providing essential nutrients for the growth of brain tumor.^176^, ^177^, ^178^, ^179^ Interestingly, brain CSCs can generate blood vessels by transdifferentiation into ECs and pericytes.^180^, ^181^, ^182^

## 6 BRAIN CSCs–IMMUNE SYSTEM INTERACTIONS

**FIGURE 4:**
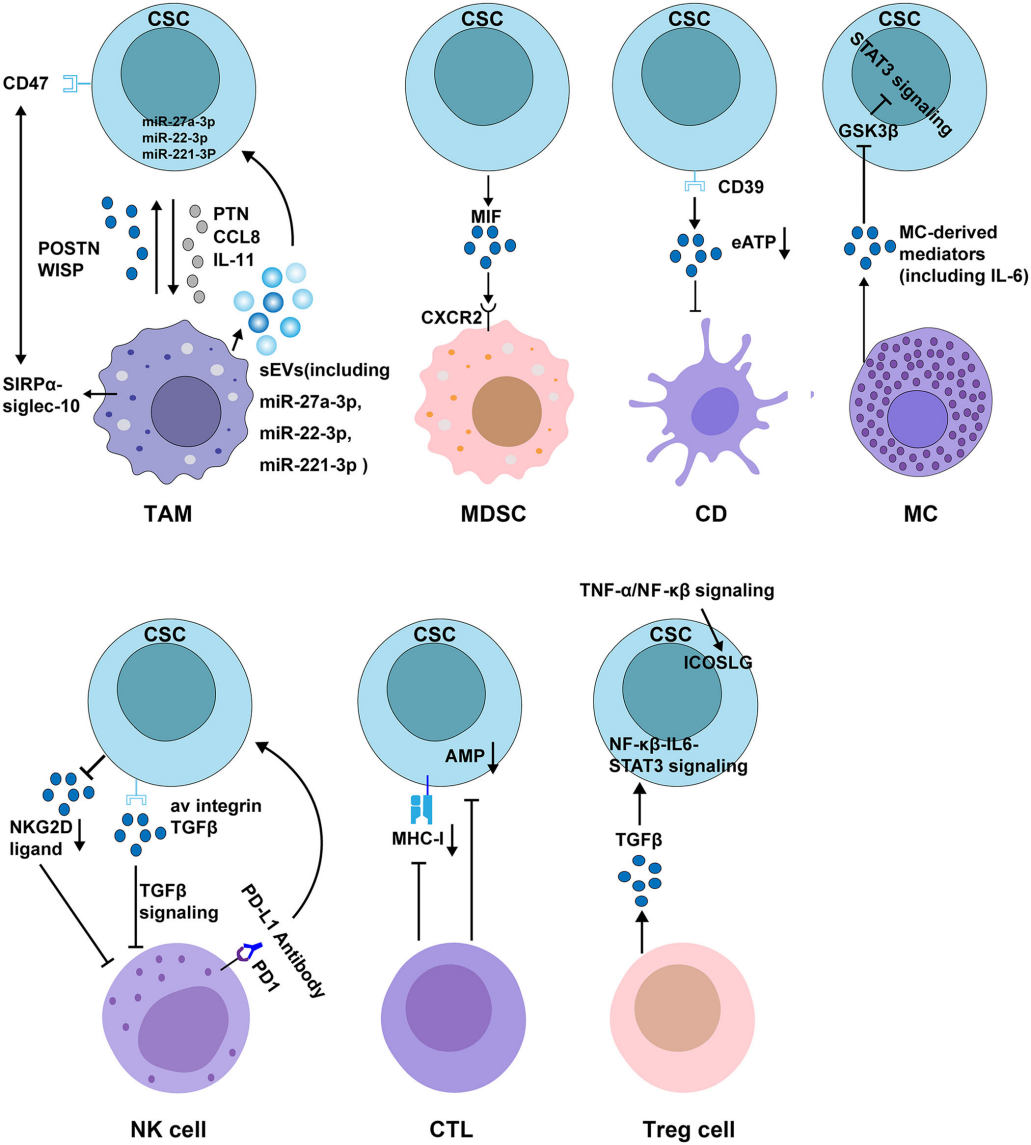
Associations between CSCs and immune cells. Reciprocal communication between CSCs and infiltrating immune cell populations in the tumor microenvironment is a key factor that simultaneously induces the formation of CSCs and reprograms the immune response, thereby facilitating immune evasion by the tumor. Biological factors known to influence interactions between brain CSCs and immune cells are shown. TAM, tumor‐associated macrophage; MDSC, myeloid‐derived suppressor cell; DC, dendritic cell; MC, mast cell; NK, natural killer; CTL, cytotoxic T lymphocyte; Treg, regulatory T cell; MHC‐I, major histocompatibility complex class I; APM, antigen‐processing machinery.

Studies have begun to clarify the relationship between brain CSCs and immune cells.^183^, ^184^ CSCs can indirectly promote tumor development by impairing immune surveillance within the tumor microenvironment. In addition, CSCs themselves can evade the immune system by altering their immunogenicity to avoid immune‐mediated rejection in vivo. In this section, we will discuss the interaction between brain CSCs and immune cells (Figure 4).

### 6.1 CSCs and tumor‐associated macrophages

In studies of brain CSCs and immunity, tumor‐associated macrophages (TAMs) are one of the most well‐studied immune cell types. In the brain tumor TME, TAMs include tissue‐resident microglia and bone marrow‐derived infiltrating macrophages^185^ that aggregate in perivascular and necrotic areas in response to ischemia.^186^, ^187^ On one hand, GSCs‐secreted periostin (POSTN) promotes TAM recruitment and supports the tumor‐promoting M2 subtype.^188^ WISP1, which is preferentially expressed and secreted by GSCs, stimulates M2 TAM polarization through the integrin α6β1–Akt pathway.^117^ On the other, TAMs produce pleiotropin (PTN) in a reciprocal manner, which stimulates the maintenance of GSCs through the receptor PTPRZ1, thereby promoting the malignant growth of GBM.^189^, ^190^ TAM also produces chemokine (C‐C motif) ligand 8 (CCL8), which promotes GBM cell invasion and stemness through ERK1/2 signaling.^191^ Further study of interaction found that IL‐11 secreted by GBM‐associated microglia/macrophages can activate STAT3–MYC signaling in GBM cells, thereby inducing stem cell states that confer enhanced tumorigenicity and resistance to the standard‐of‐care chemotherapy TMZ.^192^ Small extracellular vesicles released by TAMs were also found to transfer miR‐221‐3p, miR‐22‐3p, and miR‐27a‐3p to GSCs, and these miRNAs promoted several mesenchymal characteristics of proneural GSCs by targeting CHD7, in turn promoting chemoresistance of GSCs.^193^ However, in addition to the classical role of TAMs in promoting tumor growth, increasing evidence shows that these cells have antitumor activity in the immunosuppressive tumor microenvironment. For example, CD47 has been identified as a do not eat me signal in lung and pancreatic cancers; CD47‐expressing lung and pancreatic CSCs escape phagocytosis of macrophages.^194^, ^195^ Given that CD47 is also highly expressed on GSCs and is associated with poor clinical outcomes, it is also possible that GSCs escape macrophage clearance. Treatment with an anti‐CD47 antibody led to increased macrophage‐mediated phagocytosis of glioma cells and GSCs and had no obvious side effects on normal cells.^196^ However, the efficacy and mechanism of this treatment need to be further explored in vivo.

### 6.2 CSCs and myeloid‐derived suppressor cells

Myeloid‐derived suppressor cells (MDSCs) are myeloid‐derived immature cells with immunosuppressive capabilities. Immunosuppressive MDSCs are found in the brains of GBM patients and are in close proximity to self‐renewing CSCs. Secretion of migration inhibitory factor (MIF) by CSCs promotes MDSC‐mediated immunosuppression in a CXCR2‐dependent manner, thereby promoting GBM immune evasion.^197^ Another study showed that GSCs‐derived exosomes inhibited T‐cell immune responses by acting on monocyte maturation rather than directly interacting with T cells.^198^

### 6.3 CSCs and dendritic cells

Dendritic cells (DCs) capture, process, and present antigens to T lymphocytes to generate tumor‐specific immune responses. GSCs upregulate CD39 expression, which in turn reduces the extracellular ATP concentration around GSCs to maintain an immunosuppressive microenvironment.^199^ The combination of doxorubicin and CD39 inhibition increases the extracellular ATP concentration around GSCs, thereby recruiting DCs to phagocytose damaged GSCs and activating T cells to kill target cells.^199^ However, studies on the interaction of GSCs with DCs have largely focused on developing vaccines.^200^, ^201^, ^202^, ^203^

### 6.4 CSCs and mast cells

Mast cells (MCs) are granulocytes that play a crucial role in various inflammatory conditions, including cancer. Currently, the role of MCs in cancer is still controversial, their support or inhibition of tumor progression depends on the type and malignant degree of the cancer.^204^ MCs infiltrate human and mouse glioma in response to multiple signals in a glioma grade‐dependent manner.^205^ Further studies found that MCs activated by glioma cells reduce the proliferation, migration and self‐renewal capacity of glioma cell, and decrease the expression of stemness markers but in turn the differentiation of promote glioma cell.^206^ Specifically, MCs exert these effects via down‐modulation of GSK3β expression and suppression of STAT3 activation.^206^

### 6.5 CSCs and natural killer cells

Natural killer (NK) cells are a subtype of cytotoxic lymphocytes that plays an important role in the innate immune system and can directly identify and kill cancer cells. NK cells have been found to recognize and kill GBM cells with stem‐like properties.^207^ This targeted killing is related to the fact that GSCs do not express protective HLA class I molecules but express various ligands of activating NK receptors that trigger optimal NK cell cytotoxicity.^207^ IDH‐mutant GSCs were found to have reduced NK group 2D (NKG2D) ligand expression and were thus resistant to NK cell‐mediated lysis. Decitabine‐mediated hypomethylation increases NKG2D ligand expression in IDH‐mutant GSCs and restores NK‐mediated killing of IDH‐mutant GSCs.^208^ One study has established that GSCs are more susceptible to NK cell lysis than differentiated cells due to killer immunoglobulin‐like receptor‐human leukocyte antigen ligand mismatch and activation receptor–ligand interactions.^209^ Another study found that GSCs promote NK cell dysfunction by regulating the αv integrin/TGF‐β axis. Targeting the αv integrin/TGF‐β axis enhances NK cell killing of GSCs and suppresses tumor growth.^210^ In addition, blocking the PD‐1/B7H1 pathway encourages mouse NK cells to kill GSCs, and PD‐1‐inhibited NK cells can be a viable approach for immunotherapy against GBM.^211^

### 6.6 CSCs and tumor‐infiltrating lymphocytes

Cytotoxic T lymphocytes (CTLs) are key players in cancer immunotherapy. CTLs mediate tumor cell killing by recognizing major histocompatibility class I (MHC‐I) molecules on the tumor cell surface. However, abnormalities in MHC‐I expression are frequently seen in various malignancies.^212^, ^213^ Downregulation or deficiency of the expression of MHC‐I molecules and antigen processing machinery (APM) components in GSCs reduces T‐cell function and increases evasion of systemic immune surveillance.^214^ Increased expression of MHC‐I and APM components enhanced CTL‐mediated GSCs killing and the antitumor effect of a tumor lysate vaccine.^214^ GSCs can also indirectly influence brain tumor growth by regulating the activity of regulatory T cells (Tregs). Tregs are T cells with immunosuppressive effects that not only inhibit effector T cells activation by presenting surface antigens^215^ but also suppress other immune cells functions by secreting cytokines (TGF‐β, IL‐10, etc.) and induce other T cells to transform into Tregs.^216^, ^217^ Studies have shown dense infiltration of Tregs in glioma, where they induce the expression of the stemness‐related genes CD133, nestin, SOX2, and so on by the NF‐κB‐IL6‐STAT3 signaling pathway, thereby increasing cancer stemness and promoting tumor growth.^210^ Blockade of the IL‐6 receptor with tocilizumab inhibits Treg‐induced cancer stemness and tumor growth in a glioma xenograft model.^210^ Furthermore, Iwata et al.^218^ found that inducible T‐cell co‐stimulator ligand (ICOSLG) was preferentially upregulated in mesenchymal GSCs in a TNF‐α/NK‐kB‐dependent manner. The expression of ICOSLG in mesenchymal GSCs mediates Treg cell expansion and IL‐10 production, thereby promoting GBM progression. Knockout of ICOSLG significantly reduced GBM tumor growth in immunocompetent mice.^218^

## 7 THERAPIES TARGETING CSCS IN BRAIN

**FIGURE 5:**
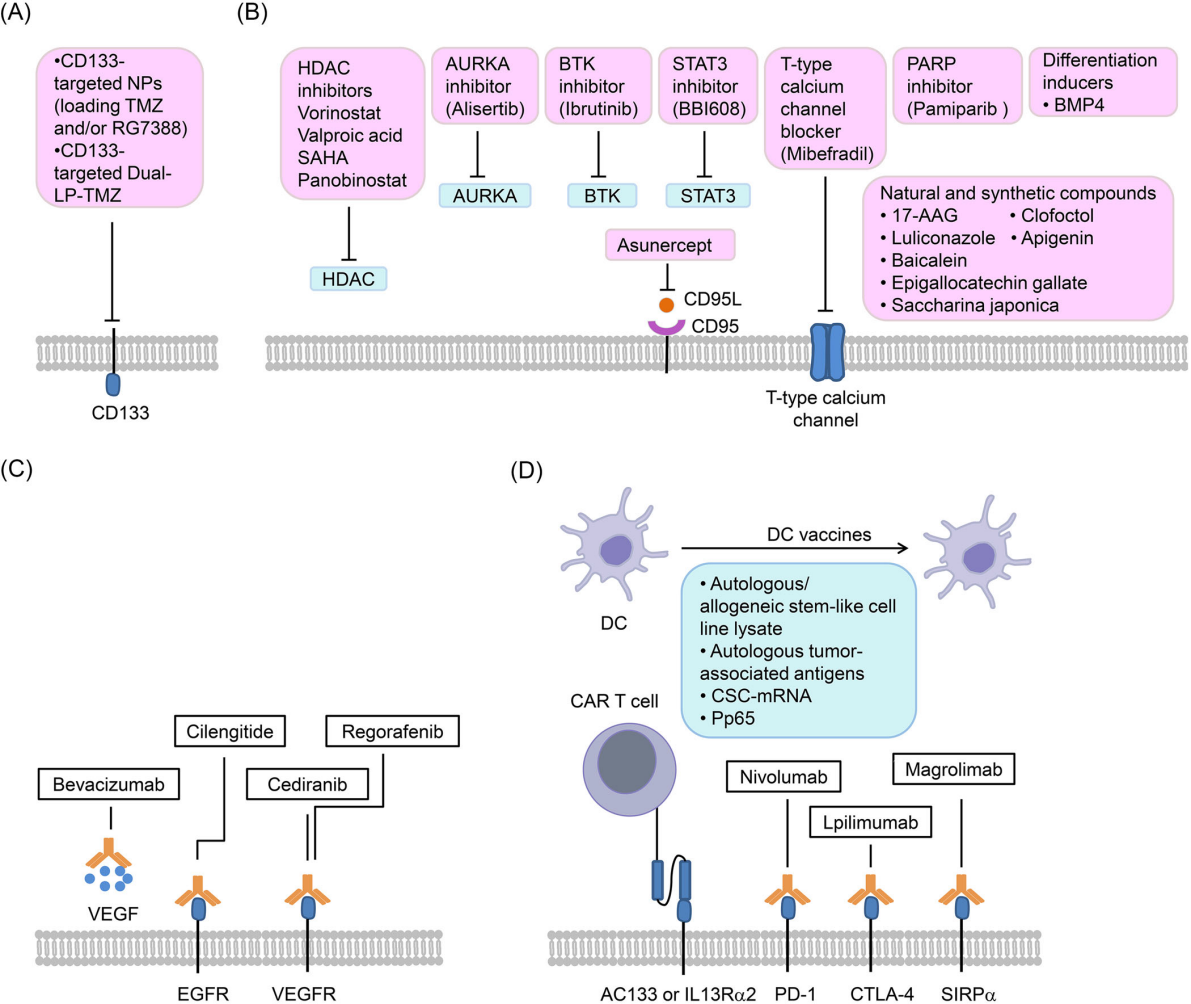
Proposed therapeutic approaches to target CSCs. Novel approaches to target CSCs, as well as various available CSC‐based therapies that have the potential to be translated for use in the clinical treatment of brain tumors are shown. These approaches include: (A) targeting CSC markers, (B) Targeting vascular niche of CSCs, (C) targeting intrinsic and extrinsic regulators of CSCs, (D) CSC‐directed therapies. CAR, chimeric antigen receptor; NPs, polymer‐micellar nanoparticles (consisting of poly(styrene‐b‐ethylene oxide) (PS‐b‐PEO) and poly(lactic‐co‐glycolic) acid (PLGA)); Dual‐LP‐TMZ, dual‐targeting immunoliposome encapsulating TMZ; DC, dendritic cell.

In order to obtain more effective treatments, novel approaches to target brain CSCS are underway. These therapeutic approaches include targeting CSC surface markers, targeting intrinsic and extrinsic regulators of CSCs and targeting immunotherapy of CSCs (Figure 5 and Table 2).

**TABLE 2:** Clinical trials targeting CSCs.

Drug name	Target	Combination	Phase (O‐III)	Sample size	ClinicalTrials.gov identifier	Current status
Alisertib	AURKA	Hyperfractionated radiation therapy + stereotactic radiosurgery	Phase I	17	NCT02186509	Completed, Has results
BBI608	STAT3	TMZ	Phase I/II	34	NCT02315534	Completed, Has results
Pamiparib	PARP1	TMZ + radiation	Phase I/II	116	NCT03150862	Completed, Has results
Asunercept	CD95L		Phase I	10	NCT02853565	Completed
Regorafenib	Angiogenesis	Lomustine	Phase II	119	NCT02926222	Completed, Has results
Bevacizumab	VEGF	Standard radiotherapy + durvalumab	Phase II	159	NCT02336165	Completed, Has results
Bevacizumab	VEGF	GM‐CSF + cyclophosphamide + ERC1671	Phase II	84	NCT01903330	Recruiting
Allogeneic GSC lysate DC vaccine	Allogeneic GSC lysate	TMZ, field radiation	Phase I	39	NCT02010606	Completed
Autologous GSC lysate DC vaccine	Autogeneic glioma stem‐like cells (A2B5+) lysate	Surgery + chemotherapy + radiotherapy	Phase II	100	NCT01567202	Recruiting
AV‐GBM‐1	Autologous tumor‐associated antigens		Phase II	55	NCT03400917	Active, not recruiting
Tumor stem cell derived mRNA‐ transfected DC vaccine	Tumor stem cell‐derived mRNA		Phase I/II	20	NCT00846456	Completed
ATTAC‐II	pp65	Td + saline	Phase II	175	NCT02465268	Recruiting
IL13Ra2 CAR T	IL13Ra2	Ipilimumab + nivolumab	Phase I	60	NCT04661384	Recruiting
Nivolumab	PD‐L1	Ipilimumab	Phase III	529	NCT02017717	Active, not recruiting, Has results
Nivolumab	PD‐L1	TMZ + radiotherapy	Phase III	560	NCT02617589	Completed
Nivolumab	PD‐L1	TZM + radiotherapy	Phase III	716	NCT02667587	Active, not recruiting, Has results
Nivolumab and ipilimumab	PD‐L1 and CTLA‐4	TMZ	Phase I	32	NCT02311920	Completed
Nivolumab, ipilimumab, and bevacizumab	PD‐L1, CTLA‐4, and angiogenesis	Hypofractionated stereotactic irradiation	Phase I	22	NCT02829931	Completed
PNOC025	CD47		Phase I	24	NCT05169944	Recruiting

TMZ, temozolomide; GM‐CS, granulocyte‐macrophage colony‐stimulating factor; Td, tetrodotoxin. Data sources—ClinicalTrials.gov (https://clinicaltrials.gov/ct2/home).

### 7.1 Targeting CSC surface markers

The role of CD133 as a brain CSC marker has been extensively studied. Lentivirus‐mediated silencing of CD133 in human GBM neurospheres has been reported to impair neurosphere cell self‐renewal and tumorigenicity,^219^ implying that CD133 can be used as a therapeutic target in GBM. In a study of CD133^+^ xenograft‐carrying immunodeficient mice, treatment with carbon nanotubes conjugated to an anti‐CD133 monoclonal antibody followed by near‐infrared laser irradiation efficiently targeted and destroyed GBM CSCs in vitro and in vivo.^220^ Polymer‐micellar nanoparticles (NPs) composed of poly(lactic‐co‐glycolic) acid and poly(styrene‐b‐ethylene oxide) were developed by a double emulsion technique loading TMZ and/or idasanutlin, and these NPs targeted the CD133 antigen expressed on the surfaces of GSCs and killed a subpopulation of CSCs.^221^ In addition, a dual‐targeting immunoliposome encapsulating TMZ (Dual‐LP‐TMZ) was developed by using angiopep‐2 and an anti‐CD133 monoclonal antibody for BBB transcytosis and specific delivery to GSCs, respectively. Dual‐LP‐TMZ increased the cytotoxicity of GSCs in vitro and significantly decreased brain tumor size in vivo.^222^ Notably, our current understanding of brain CSCs is largely influenced by NSC biology. NSCs and CSCs share many identified markers, such as CD133 and integrin 6, so eradication of CSCs in brain tumor by targeting these markers may also lead to a reduction in NSCs or neural tissue cells, which may affect brain function.

### 7.2 Targeting intrinsic and extrinsic regulators of CSCs

Drugs that target brain CSCs‐associated self‐renewal pathways are not discussed in this section because they have been thoroughly reviewed elsewhere.^223^, ^224^, ^225^, ^226^ At present, many inhibitors targeting the regulation of CSC factors have been successfully developed and applied in clinical research. Vorinostat, a potent oral HDAC inhibitor, has been shown to be a potent sensitizer to standard radiotherapy and TMZ in preclinical studies and has the potential to increase the cytotoxic activity of radiotherapy and TMZ in GBM.^227^, ^228^ Clinical trials of HDAC inhibitors, including valproic acid, SAHA, and panobinostat, are ongoing in patients with various cancers. Alisertib (MLN8237), an oral selective AURKA inhibitor, synergizes with TMZ to inhibit the growth of GBM and increase the effect of ionizing radiation on GBM tumor stem‐like cells.^229^ A phase I clinical trial of alisertib combined with fractionated stereotactic re‐irradiation therapy (FSRT) (NCT02186509) showed that FSRT combined with alisertib is safe and well tolerated in recurrent high‐grade glioma.^230^ BTK is highly expressed in clinical glioma samples, and is a prognostic marker and molecular therapeutic target for glioma. Preclinical data obtained from GBM cell lines and primary tumors showed that ibrutinib (a targeted BTK blocker) has strong antitumor activity.^112^, ^231^ A recently completed phase I‐II clinical trial (NCT02315534) tested the combination of BBI608 (a STAT3 inhibitor) and TMZ in patients with relapsed or advanced GBM with encouraging results. The combination of BBI608 with TMZ has the potential to target GSCs. The DNA repair enzyme PARP1 is highly expressed in primary GBM patient samples and expressed at significantly lower levels in normal neurons from controls and GBM patients.^232^ Inhibition of PARP1 restores GSCs sensitivity to TMZ,^233^ enhances glioma‐initiating cell sensitivity to radiation, and inhibits glioma‐initiating cell growth, self‐renewal, and DNA damage repair. In vivo combination treatment with a PARP inhibitor and radiation attenuated radiation‐induced glioma‐initiating cell enrichment and suppressed the tumor‐initiating CSC phenotype.^234^, ^235^ In preclinical studies, the combination of pamiparib (PARP1 inhibitor) and TMZ overcame TMZ resistance and showed significant tumor suppression.^236^ The combination of pamiparib and TMZ is currently being evaluated in a clinical trial (NCT03150862).

CD95 maintains the stem‐like and nonclassical EMT programs of GBM,^125^ and a phase I clinical trial (NCT02853565) in newly diagnosed Asian patients showed that asunercept (which selectively binds to CD95 ligand (CD95L) and disrupts CD95/CD95L signaling) added to standard radiotherapy/TMZ was safe and well tolerated, with encouraging efficacy when administered at 400 mg/week.^237^ Mibefradil is a selective T‐type calcium channel blocker that inhibits the growth of glioma cells and increases the sensitivity of GSCs to chemotherapy. A phase I clinical trial showed that mibefradil was well tolerated in patients with high‐grade glioma.^238^

Various small molecules (such as 17‐AAG) and analogs of naturally occurring compounds (such as clofoctol and luliconazole) are able to suppress brain CSCs growth. Apigenin (a flavonoid compound present in a variety of edible plants and health foods),^239^ baicalein (an aromatic tropolone found in *Chamaecyparis taiwanensis*),^240^ epigallocatechin gallate (a bioactive polyphenol in green tea)^241^ and saccharina japonica (a member of the Phaeophyceae (brown algae) family)^242^ have all been separately demonstrated to target GSCs by attenuating HIF‐1α‐mediated glycolysis, targeting Nrf2 regulation, decreasing P‐glycoprotein inhibition and degrading EGFR/EGFR variant III, respectively.

### 7.3 Targeting vascular niche of CSCs

Conventional approaches for cancer therapy have been developed without highlighting the tumor microenvironment, so a new focus targeting cancer therapy is to limit cancer progression by targeting the unique microenvironment of CSCs. Disruption of the perivascular niche may be a key approach to target CSCs. Directly targeting ECs, which make up tumor blood vessels, is an alternative approach for destabilizing CSCs function.^243^ Numerous clinical trials evaluating antiangiogenic therapies have been reported with mixed results (75 clinical trials, 53 of which involved bevacizumab). Most antiangiogenic therapies (such as cilengitide, cediranib, or bevacizumab) have not demonstrated significant improvement in OS and quality of life in patients.^244^ This limited efficacy may be related to VEGF‐independent angiogenesis, the inefficient delivery of antiangiogenic factors to tumor.^245^ Various attempts have been made to develop innovative therapies based on antiangiogenic factors that target angiogenesis via different mechanisms of action. Recently, regorafenib (NCT02926222), a kinase inhibitor that regulates neoangiogenesis, has shown encouraging results when used as monotherapy for recurrent GBM.^246^

Recently, antiangiogenic therapies have been proposed in combination with immunotherapies, such as the Gliovac study (vaccine‐based therapy plus bevacizumab) (NCT01903330) and Durvalumab (immune checkpoint inhibitors (ICIs) plus bevacizumab) (NCT02336165). Although there have not yet been any successful phase III trials of combined anti‐VEGF therapy and immunotherapy for brain tumors, the combination strategies targeting brain TME remains an attractive approach.

### 7.4 CSC‐directed immunotherapy

The brain tumor microenvironment is highly immunosuppressive because of its weak immunogenicity and the immunosuppressive properties of many cells, including CSCs, cancer cells, and immunosuppressive tumor‐infiltrating immune cells.^247^, ^248^ Therefore, immunotherapy may be a promising avenue for the treatment of brain tumor. Approaches include the use of CSC‐derived materials as antigen sources to produce DC vaccines, using genetically engineered chimeric antigen receptor (CAR) T cells to generate CSC‐specific T cells, and using monoclonal antibodies with direct inhibitory effects and/or the ability to induce antibody‐dependent cellular cytotoxicity to target CSC‐associated cell surface markers.

#### 7.4.1 DC vaccines

CD133 is a tumor stem‐like cell antigen that may elicit strong immune responses in patients with malignant glioma.^249^ Phase I studies have confirmed that DC vaccines with GSC‐related antigens are safe in patients with recurrent glioma.^249^ A phase I study of an autologous DC vaccine pulsed with allogeneic stem‐like cell line lysate in patients with newly diagnosed or recurrent GBM has produced promising results (NCT02010606). In the study, cytotoxic T‐cell reactions were induced in some vaccinated patients.^250^ In addition, the efficacy of autologous DCs loaded with autogeneic glioma stem‐like cells (A2B5^+^) administered as a vaccination in adults with GBM multiforme is being assessed in a phase II study (NCT01567202). Other groups are utilizing patient surgical specimens to culture GSCs and train autologous DCs (NCT03400917). In addition to GSC lysate, mRNA from patient‐derived GSCs can be used to generate personalized vaccines. One phase I trial (NCT00846456) demonstrated the safety of this approach as well as a nearly 2.9 times longer progression‐free survival (PFS) than that achieved with matched controls.^251^ Additionally, the phosphoprotein pp65, a product of human cytomegalovirus, is an intriguing DC vaccine target. In a small clinical trial, a pp65 DC vaccine and a dose‐enhanced TMZ cycle were combined, and pp65‐specific cellular responses were evaluated (NCT00639639). Preliminary findings showed that the OS was more than doubled and the PFS was tripled when compared with historical controls.^252^ Of note, four patients remained progression‐free 59–64 months after diagnosis. Phase II of this trial (NCT02465268) is currently ongoing.

#### 7.4.2 CAR T cells

CAR T cells targeting several brain CSCs ‐specific antigens have been developed, such as CAR T cells targeting the CD133 epitope AC133. These AC133‐specific CAR T cells can recognize and eradicate patient‐derived AC133(+) GSCs and improved OS in treated mice.^253^, ^254^ In addition, CAR T cells can also be used to target non‐CSC‐specific peptides that are also upregulated in brain CSCs, such as IL‐13 receptor α2 (IL13Rα2). Intracranial delivery of the IL13Rα2‐directed CD8^+^ CAR T‐cell clones into the resection cavity in three patients with recurrent disease was well tolerated.^255^ A phase I clinical trial of L13Rα2‐CAR T cells as treatment for patients with leptomeningeal disease from MB, GBM or ependymoma is ongoing (NCT04661384).

#### 7.4.3 Antibodies

Antibodies have been utilized for cancer treatment for two decades and have greatly contributed to tumor immuno‐oncology by inducing direct cell killing and regulating cellular immune response.^256^ Monoclonal antibodies called ICIs have been developed to enhance the activity of T cells by blocking negative regulatory pathways mediated by surface receptors known as immune checkpoints.^257^, ^258^ Among the various immune checkpoints being studied, programmed cell death protein 1 (PD‐1) and cytotoxic T lymphocyte associated antigen 4 (CTLA‐4) are the most commonly studied molecules.^259^ The CheckMate‐143 (NCT02017717) phase I clinical trial evaluates the effectiveness and safety of nivolumab (PD‐1 inhibitor) alone or in combination with ipilimumab (CTLA‐4 inhibitor) in recurrent GBM. Interestingly, nivolumab monotherapy has a higher median OS than nivolumab plus ipilimumab; and moreover, higher adverse events is observed only when the combination is administered. These findings suggest a requirement for further investigation. In the CheckMate‐143 phase III clinical trial, the use of the PD‐1 antibody nivolumab does not improve survival compared with the VEGF inhibitor bevacizumab.^260^ Of note, nivolumab may be beneficial for patients with MGMT promoter methylation and without baseline corticosteroid use.^260^ Therefore, subsequent studies evaluate nivolumab in patients without and with methylated MGMT promoter.^261^ The results from the CheckMate‐498 Phase III trial showed that nivolumab does not confer a survival advantage over TMZ in patients without MGMT promoter methylation (NCT02617589).^261^ Similarly, in the checkmate‐548 trial assessing standard‐of‐care plus nivolumab or placebo in patients with newly diagnosed GBM with MGMT promoter methylation, no significant improvement in survival is observed (NCT02667587).^262^ However, it is worth noting that patients with PD‐L1 expression levels greater than 5% in this study show a trend toward improved PFS. Additionally, clinical trials (NCT02311920, NCT02829931) assessing ipilimumab therapies have been completed, but the results have not yet been published.

In addition to CTLA‐4 and PD‐1 blockade, monoclonal antibodies targeting other checkpoint molecules, such as CD47 antibody, have been investigated in clinical trials for brain tumors. It was previously found that anti‐CD47 antibody treatment increased the phagocytosis of GSCs by macrophages.^196^ Therefore, using anti‐CD47 monoclonal antibodies to enhance phagocytosis may be another promising strategy to target CSCs. Currently, the safety of magrolimab, a first‐in‐class anticancer therapeutic agent targeting the CD47‐signal receptor protein‐alpha (SIRP‐alpha) axis, is being tested in children and adults with recurrent or progressive malignant brain tumors (NCT05169944).

At present, the efficacy of ICIs in brain tumors has been disappointing, possibly related to the excessive presence of MDSCs and Tregs and insufficient tumor‐infiltrating lymphocytes, leading to poor downstream checkpoint‐blocking effects.

### 7.5 Therapeutic resistance of CSCs

Single‐cell sequencing and epigenetic analysis methods reveal new insights into CSCs diversity, plasticity, dynamic cell states, redundant escape mechanisms, and enhanced adaptability to harsh microenvironments, which are critical to understanding CSCs resistance to current cancer therapies. Currently, conventional therapy focuses on dividing cells, and CSCs have been thought to be in a dormant or quiescent phase after tumor formation.^263^ The relative quiescent of CSCs enable CSCs to survive chemotherapy and radiotherapy, evade the immune system, and promote tumor progression or recurrence. For example, quiescent CSCs are resistant to TMZ treatment in a genetically engineered mouse model of glioma. However, after diphtheria toxin‐mediated ablation of CSCs, tumor sensitivity to TMZ therapy is enhanced.^9^ In targeted therapy with response receptor tyrosine kinase inhibitors, GSCs can reversibly transition to a slow cycling, quiescent state through high expression of histone lysine demethylase 6A /B and Notch signaling, thus transiting GSCs to a less sensitive state in the treatment response.^264^ These slow‐cycling cells are identified in GBM patients before treatment, supporting the notion that quiescent CSC populations are preexisting, and show cellular plasticity through their ability to switch between cellular states.

On the other hand, preclinical studies have demonstrated that CSCs can lead to radiotherapy and chemotherapy resistance by activating DNA damage repair mechanisms.^265^ Many of the signaling pathways essential for stemness maintenance of CSCs also promote DNA damage repair. For example, NOTCH signaling mediates radioresistance in GSCs through upregulation of the pro‐survival pathways PI3K/AKT and Bcl‐2.^266^ Other resistance mechanisms include enhanced protection against reactive oxygen species and upregulation of drug efflux pumps.^267^, ^268^

In conclusion, antiproliferative treatment regimens lead to eradication of most dividing tumor cells, but leave quiescent CSCS resistant to treatment. These cells replenish new tumor cells by producing transient populations of hyperproliferative cells that act as a reservoir to repopulate the tumor.

## 8 CLINICAL IMPLICATIONS

Despite advances in the understanding of brain CSCs, many factors remain to be studied, including the origin cells of brain CSCs and the factors driving brain CSCs phenotypic plasticity, and the lack of knowledge regarding these factors complicates the identification and eradication of brain CSCs. As already discussed, it is difficult to identify the cell population responsible for malignant transformation in brain tumors. Several studies have discovered that brain CSCs originate from NSCs, mature glial cells, or restricted neural progenitor cells. However, in performing both CSC and tumor cell‐of‐origin studies, it has been found that CSCs can also originate through the dedifferentiation of bulk tumor cells. Indeed, any cell in the glial lineage can be the target of oncogenic transformation and obtain common CSC features associated with tumorigenesis by activating various cell‐type‐specific signaling pathways, which contributes to the phenotypic and genetic heterogeneity of brain tumors. Improving the understanding of CSC plasticity and enhancing the recognition of subpopulations of non‐CSC that can be converted to CSCs will help to dissect tumor heterogeneity and find CSC‐specific therapeutics.

At present, a major limitation of therapies against brain CSCs is their target, which primarily consists of stemness‐associated intracellular signaling and/or transcriptional factors that are shared with NSCs. Additionally, existing therapies targeting signaling in brain CSCs lack specificity, emphasizing the need for the design of new inhibitors. Therefore, it is crucial to search for new potential biomarkers that can easily be translated into the clinic. The identification of putative new CSC biomarkers and novel and unique pathways that are active in this subset of cells have significant clinical relevance. In particular, targeting cell surface CSC markers is a promising strategy as they represent accessible targets for monoclonal antibodies or drugs, which allows easy regulation of CSC signaling.

Numerous studies have shown that brain CSCs can promote immune evasion and enhance the expansion of protumorigenic immune phenotypes.^117^, ^216^, ^217^ Multiple approaches targeting the interactions between the immune system factors and CSCs are actively being studied. In addition, various anti‐CSC immunotherapies are presently in clinical development. Research into the interplay between tumor microenvironments and brain CSCs has shown that phenotypes of stem cells vary considerably within the tumor and that brain CSCs subpopulations may have differential sensitivity to immunotherapeutic approaches.^269^ These findings have provided a foundation for seeking multiple modalities of immunotherapey based on the relative permissiveness of brain CSCs populations. DC vaccination may be the most promising form of immunotherapy against high‐grade glioma. Approximately 30 clinical trials investigating DC vaccines against high‐grade glioma are ongoing.^270^ Although preclinical and clinical studies have demonstrated that DC vaccines can train cytotoxic T cells to target brain CSCs without deleterious off‐target effects, these vaccines are not approved by the US FDA. At present, there are not yet drugs available to eliminate this cancer‐driving cell population, but the numerous strategies being pursued to target brain CSCs are promising and worth investigating.

## 9 CONCLUSIONS

To date, there are no effective therapies that have substantially impacted the clinical outcomes of patients with malignant brain tumors. However, the identification of CSCs and their crucial role in tumor progression and resistance to therapy has opened up new avenues for the discovery of novel therapeutic targets. Researchers have been investigating the potential targets of antitumor drugs targeting CSCs by studying brain CSCs surface markers, such as CD44 and CD133. Additionally, intracellular regulatory factors that are associated with the proliferation of brain CSCs and the maintenance of their stem‐like properties have also been successively elucidated. Aberrant expression of these intracellular regulatory factors is closely associated with tumor relapse and metastasis. Investigating these factors and their inhibitors provides a theoretical foundation and impetus for the development of antibrain tumor drugs targeting brain CSCs. Despite the maturation of theories related to brain CSCs, several questions remain controversial and require resolution to systematically and effectively translate mechanistic insights into novel therapeutic interventions. First, the effectiveness of isolation and identification of CSCs using surface markers are controversial. This is because these markers are not exclusive to the CSC subpopulation of brain tumor cells, and brain CSCs and normal stem cells share similar markers, making it difficult to distinguish them from each other during treatment. Therefore, it is challenging to demonstrate a satisfying targeting effect. Further studies are necessary to investigate more specific markers and develop more targeted therapies against brain CSCs. Second, cell‐intrinsic molecular mechanisms that regulate brain CSCs have been identified, which at least partially overlap with the mechanisms responsible for maintaining normal brain homeostasis, thus, not all the regulatory factors that contribute to CSCs are suitable targets for therapeutic intervention. Third, stromal and immune cells within CSC niches play a critical role in establishing a structural and functional barrier to protect CSCs against external attack; however, most studies investigating the interaction between CSCs and their niche mainly rely on 2D cell culture, which may not accurately represent the complex 3D microenvironment of CSCs in vivo. Finally, the majority of oncological investigations related to CSCs are conducted in immune‐deficient mice, which lack the biological complexity associated with the infiltration of immune cells and their specific actions in the TME in human patients.

In summary, brain CSCs have been identified as critical drivers of the recurrence and metastasis that are major lethal causes of brain cancer patients. Research in this area has focused on exploiting the therapeutic potential of molecular mechanisms regulating stemness, but rigorous testing and validation of targets will be necessary. As investigations into stemness‐associated signal transduction continue, we anticipate that CSC theories will become more refined and provide a more authoritative basis for drug discovery and clinical treatment, thereby improving the survival and efficacy of brain tumor patients.

## AUTHOR CONTRIBUTIONS

S. Y. L. wrote the manuscript. K. S. L. participated on the writing of the manuscript, corrected the manuscript, and contributed with critical discussion. Q. L. conceived the original idea, corrected and finalized the manuscript, and contributed with critical discussion. All authors read and approved the final manuscript.

## CONFLICT OF INTEREST STATEMENT

The authors declare no conflict of interest.

## ETHICS STATEMENT

Not applicable.

## Data Availability

Not applicable.
